# Supplementary material for: Content-rich biological network constructed by mining PubMed abstracts
Source: BMC Bioinformatics. 2004 Oct 8;5:147. doi: 10.1186/1471-2105-5-147 (PMC528731; doi:10.1186/1471-2105-5-147)
Supplement: Additional File 2 — The original results of the above study (non-essential files are deleted to keep the file size under the limit set by BMC bioinformatics). [file 1471-2105-5-147-S2.bz2 › chilibotAdditionalFile2/dip40/278ID2497053E1101/html/JUN_FOSL1.html]

 


 **JUN** and **FOSL1** 
  
Found 404 abstracts in PubMed, retrieved 40.  
 

 What does Google say? 
 PDF only 
| .edu only 

---

**Interactive relationship** (e.g. stimulation, inhibition, etc)

**Stimulatory relationship**- In summary, we demonstrate that ERK dependent Fra 1  [ **FOSL1** ]  is elevated in AP 1  [ **JUN** ]  complexes in response to asbestos fibers and is critical to the transformation of mesothelial cells.  Ref: 12414630 Cancer Res, 2002
- We demonstrate that one of these sites binds c  **Jun**  Fra1  [ **FOSL1** ]  heterodimers in vitro, but that this and the other AP 1  [ **JUN** ]  like sites are dispensable with respect to c  **Jun**  stimulated SPARC promoter activation.  Ref: 12370830 Oncogene, 2002
- In differentiated 3T3 L1 adipocytes, insulin stimulated the expression of the mRNA for the genes encoding Fra 1  [ **FOSL1** ]  >100 fold, which is a component of the AP 1  [ **JUN** ]  transcriptional complex, beta actin 6.0 fold and hexokinase II 2.4 fold.  Ref: 12393186 Biochim Biophys Acta, 2002
- Taken together, our results indicate that 1alpha, 25 dihydroxyvitamin D3, via binding to the membrane receptor annexin II, induces activation of the phos phatidylinositol 3 kinase Ras MEK extracellular signal regulated kinase 1 2 and c  **Jun**  N terminal kinase 1 signal transduction pathway resulting in increased expression of c Fos, Fra1  [ **FOSL1** ] , and c  **Jun** , and subsequently increased activator protein 1  [ **JUN** ]  DNA binding activity and gene transcription.  Ref: 12648218 J Invest Dermatol, 2003
**Neutral relationship**- An electrophoretic mobility shift assay confirmed the AP 1  [ **JUN** ]  binding activity in SMCs and showed that the protein bound to the AP 1  [ **JUN** ]  site consisted predominantly of c  **Jun** , JunD and Fra 1  [ **FOSL1** ] .  Ref: 12622720 Genes Cells, 2003
- Transfection of PC12 cells with Fra  [ **FOSL1** ]  2 induced reporter activity requiring the AP1  [ **JUN** ] , but not the Egr1 motif.  Ref: 12670703 Brain Res Mol Brain Res, 2003
- Among the immediate early genes, Fra 1  [ **FOSL1** ]  and  **Jun**  B immunoreactivities were found in Müller cells whereas c  **Jun**  and cyclin D1 immunoreactivities were found in apoptotic retinal neurons.  Ref: 12714663 Invest Ophthalmol Vis Sci, 2003
- Nuclear extracts from granulosa cells from 1 2 or 8 10 mm antral follicles bound an AP 1  [ **JUN** ]  DNA consensus sequence and complexes consisted predominantly of c  **Jun** , JunD, JunB, c Fos, and Fra  [ **FOSL1** ]  2.  Ref: 12606371 Biol Reprod< MedlineTA>Biol Reprod, 2003
- These studies demonstrate that a functional AP 1  [ **JUN** ]  site mediates MMP 2 transcription in cardiac cells through the binding of distinctive Fra1  [ **FOSL1** ]  JunB and FosB JunB heterodimers.  Ref: 12371906 Biochem J, 2003
- Here, the major cis regulatory elements that control IFN gamma inducible NOS2 gene transcription in human lung epithelial cells are identified as composite response elements that bind signal transducer and activator of transcription 1 STAT 1 and activator protein 1  [ **JUN** ]  AP 1  [ **JUN** ] , which is comprised of c Fos, Fra  [ **FOSL1** ]  2, c  **Jun** , and JunD.  Ref: 12788789 Am J Physiol Lung Cell Mol Physiol, 2003
- Stimulation induces de novo synthesis of the AP 1  [ **JUN** ]  members JunD and Fra  [ **FOSL1** ]  2, which bind to CLE0.  Ref: 12354764 J Biol Chem, 2002
- c Fos and Fra 1  [ **FOSL1** ] , well known immediate early gene products, are members of the AP 1  [ **JUN** ]  family.  Ref: 12622723 Genes Cells, 2003
- The oncogene dependent transcriptional activation involves the cooperation between both Raf dependent and Raf independent pathways and is mediated by an AP 1  [ **JUN** ]  site within the fra 1  [ **FOSL1** ]  first intron, which becomes stably occupied by a transcriptionally active Fra 1  [ **FOSL1** ]  containing complex in ras transformed cells.  Ref: 12773579 Mol Cell Biol, 2003
- In rat, the transcription factor activating protein 1 AP 1  [ **JUN** ]  displays a circadian rhythm in its DNA binding in the pineal gland, which results from the rhythmic expression of Fra  [ **FOSL1** ]  2.  Ref: 12358739 J Neurochem, 2002
- The complexes formed with labeled AP1  [ **JUN** ]  E box oligonucleotide were reduced or supershifted with antisera to Fos family, c Fos, Fra  [ **FOSL1** ]  2, and  **Jun**  D.  Ref: 12670703 Brain Res Mol Brain Res, 2003
- From transient transfection analysis c  **Jun**  was found to strongly activate a CYP2J2 luciferase reporter construct, but cotransfection with plasmids encoding c Fos or c Fos related antigens Fra 1  [ **FOSL1** ]  and 2, abrogated reporter activity.  Ref: 12737630 Biochem J< MedlineTA>Biochem J, 2003

**Non-interactive relationship** (e.g. studied together, co-existance, homology, etc.)

- Gene disruption studies indicate that the AP 1  [ **JUN** ]  family members c  **jun** , junB, and fra1  [ **FOSL1** ]  are essential for embryonic development.  Ref: 12424143 Am J Physiol Lung Cell Mol Physiol, 2002
- Phosphorylated c  **Jun** , JunB, JunD, and Fra 1  [ **FOSL1** ] , but not c Fos, FosB, or ATF 2, are detected in the AP 1  [ **JUN** ]  DNA binding complex in cells exposed to trivalent arsenicals.  Ref: 12475910 FASEB J, 2003
- Immunoblot analyses confirmed that c  **Jun** , JunD, JunB, c Fos, Fra 1  [ **FOSL1** ] , Fra  [ **FOSL1** ]  2 and FosB immunoreactive proteins were present in whole cell extracts of all antral follicles and midluteal phase corpora lutea as well as granulosa cells isolated from different sized antral follicles.  Ref: 12606371 Biol Reprod< MedlineTA>Biol Reprod, 2003
- Hence, the antiproliferative effect of 5D seems associated to enhancement of c  **jun** , Fra  [ **FOSL1** ]  2, IGFBP3 and p21.  Ref: 12648525 J Steroid Biochem Mol Biol, 2003
- Western blotting revealed that stress for 3 hr induced significant increases in expression of the components of AP1  [ **JUN** ]  complex, c Fos, c  **Jun** , and  **Jun**  B proteins, in adrenals, without markedly affecting expression of Fos B, Fra  [ **FOSL1** ]  2, and  **Jun**  D proteins.  Ref: 12271465 J Neurosci Res, 2002
- Gene expression for Fos was suppressed at both times, while that for  **Fosl1**  and  **Jun**  was augmented at 12 h and suppressed at 48 h.  Ref: 12875578 Acta Otolaryngol, 2003
- In a Gal4 fusion assay, Pdcd4 specifically inhibited activation of c  **Jun**  and c Fos activation domains, but did not inhibit activation of JunB, JunD, Fra 1  [ **FOSL1** ] , or Fra  [ **FOSL1** ]  2.  Ref: 12802278 Oncogene, 2003
- MATERIAL AND METHODS From cDNA microarray screening of those specimens, several gene clusters, including Nos2 and the transcription factors Fos,  **Fosl1** ,  **Jun**  and Nfkb1, were identified as possible upstream regulators of Na transport protein expression.  Ref: 12875578 Acta Otolaryngol, 2003
- We found that the zinc finger region of BCL 6 interacts with c  **Jun** , JunB, and JunD proteins but does not bind c Fos or Fra  [ **FOSL1** ]  2 proteins.  Ref: 12165517 J Immunol, 2002
- Previously, we have shown that the transcriptional stimulation of SPRR1B expression by phorbol 12 myristate 13 acetate PMA is mainly mediated by a 150 94 bp enhancer harboring two critical 12 O tetradecanoylphorbol 13 acetate responsive elements TREs and by  **Jun** .Fra 1  [ **FOSL1** ]  dimers.  Ref: 12682075 J Biol Chem, 2003
- These findings strongly suggest that, in C6 cells, Fra 1  [ **FOSL1** ] ,  **Jun**  B, NFkappaB p50, and NFkappaB p52 appear to be involved in the regulation of iNOS mRNA induced by LT.  Ref: 12770614 Life Sci, 2003
- This effect was specific because palytoxin had little effect on c  **Jun** , JunB, JunD, FosB, Fra 1  [ **FOSL1** ] , or Fra  [ **FOSL1** ]  2 binding or on overall levels of transcription factor binding.  Ref: 12460732 Toxicol Appl Pharmacol, 2002
- The systemic administration of NMDA at 100 mg kg resulted in marked expression of c Fos, Fra  [ **FOSL1** ]  2 and c  **Jun**  proteins in the granule cell layers of the dentate gyrus in murine hippocampus 2 h later, followed by a significant reduction of the incorporation of 5 bromo 2 deoxyuridine in a manner sensitive to the antagonist dizocilpine 2 days after administration.  Ref: 12562521 J Neurochem, 2003
- One component of the AP 1  [ **JUN** ]  complex is the transcription factor Fra 1  [ **FOSL1** ]  fos related antigen 1  [ **FOSL1** ] , and we have demonstrated previously that insulin stimulates the expression of Fra 1  [ **FOSL1** ]  mRNA in CHO.T cells Griffiths, Black, Culbert, Dickens, Shaw, Gillespie and Tavaré 1998 Biochem.  Ref: 12197835 Biochem J, 2002
- We identified de regulated AP1  [ **JUN** ] , FRA 1  [ **FOSL1** ]  and c myc mediated transcription in cell line BSp73 ASML.  Ref: 12174879 Anticancer Res< MedlineTA>Anticancer Res, 2002
- Our data demonstrate for the first time that a combined aberrant activation of NF kappaB p50 and p65 and AP 1  [ **JUN** ]  JunD and Fra 1  [ **FOSL1** ]  in androgen independent prostate cancer cells results in deregulated IL 6 expression, suggesting a novel potential entry point for therapeutic intervention in prostate cancer.  Ref: 12727841 Cancer Res, 2003
- This sustained transcriptional response correlated with a modest sustained increase in adrenal TH AP1  [ **JUN** ]  binding, but not in the levels of Fra  [ **FOSL1** ]  2 or other fos or  **jun**  proteins.  Ref: 12538809 J Pharmacol Exp Ther, 2003
- The JNK deficient cells exhibited decreased expression of c  **Jun** , JunD, c Fos, Fra1  [ **FOSL1** ] , and Fra2.  Ref: 12665585 Mol Cell Biol, 2003
- To this end, we studied the kinetics of serum stimulated transcription of c fos, c  **jun** , fra 1  [ **FOSL1** ] , egr 1, and cyclinD1 genes, as well as the effects of sodium butyrate, an inhibitor of histone deacetylase activity, on transcription of these genes in normal REF cells and transformants E1A ras.  Ref: 12804589 Biochem Biophys Res Commun, 2003
- These results suggest that proENK mRNA expression induced by repeated nicotine administrations may be mediated by AP 1  [ **JUN** ]  proteins, such as c Fos, c  **Jun**  and Fra  [ **FOSL1** ]  2 rather than CREB via interacting to the ENKCRE 2 DNA binding domain in rat adrenal medulla.  Ref: 12269402 Life Sci, 2002
- Supershift EMSA revealed that VT preferentially induced JunB, JunD, phosphorylated c  **Jun** , c Fos, and Fra  [ **FOSL1** ]  2 binding activities of the AP 1  [ **JUN** ]  family.  Ref: 12167214 J Toxicol Environ Health A, 2002
- Under conditions of high rhodopsin availability during exposure to light, Fra  [ **FOSL1** ]  2 and, to a minor degree, FosB substitute for c Fos and enable light induced AP 1  [ **JUN** ]  activity and thus photoreceptor apoptosis.  Ref: 12766089 Invest Ophthalmol Vis Sci, 2003
- By western blot analysis, AP 1  [ **JUN** ] , such as Fra 1  [ **FOSL1** ] ,  **Jun**  B, and phospho CREB protein levels were increased by LT and translocation of NFkappaB p52 from the cytoplasm to the nucleus was increased.  Ref: 12770614 Life Sci, 2003
- Differentiation to the luteal phenotype involves a reduction in nuclear c  **Jun**  and c Fos and a predominance of JunD and Fra  [ **FOSL1** ]  2.  Ref: 12606371 Biol Reprod< MedlineTA>Biol Reprod, 2003
- Electrophoretic mobility shift assay EMSA showed that AP 1  [ **JUN** ]  and NFkappaB DNA binding activities were increased at 6 hr and these AP 1  [ **JUN** ]  and NFkappaB DNA bands increased by LT were super shifted when Fra 1  [ **FOSL1** ] ,  **Jun**  B, or NFkappaB p50 antibody was coincubated.  Ref: 12770614 Life Sci, 2003
- In CL, c  **Jun** , JunD, JunB and Fra  [ **FOSL1** ]  2 were present in DNA binding complexes and c Fos binding was not detected.  Ref: 12606371 Biol Reprod< MedlineTA>Biol Reprod, 2003
- Among the early response genes analyzed, c myc, junB, junD, c  **jun** , c fos, fosB, fra  [ **FOSL1** ] , as well as max, mad1 4, sin3, only c  **jun**  and fra  [ **FOSL1** ]  2 mRNAs were up regulated after 5D exposure.  Ref: 12648525 J Steroid Biochem Mol Biol, 2003
- Mesothelial cell transformation requires increased AP 1  [ **JUN** ]  binding activity and ERK dependent Fra 1  [ **FOSL1** ]  expression.  Ref: 12414630 Cancer Res, 2002
- Here, we demonstrate that asbestos induced mesothelial cell transformation is linked to increases in AP 1  [ **JUN** ]  DNA binding complexes and the AP 1  [ **JUN** ]  component, Fra 1  [ **FOSL1** ] .  Ref: 12414630 Cancer Res, 2002
- c Fos, c  **Jun**  and Fra  [ **FOSL1** ]  2 protein levels were persistently increased until the seventh administration.  Ref: 12269402 Life Sci, 2002
- Increased Notch1 signaling, but not Notch2, causes a dramatic down modulation of HPV driven transcription of the E6 E7 viral genes, through suppression of AP 1  [ **JUN** ]  activity by up regulation of the Fra 1  [ **FOSL1** ]  family member and decreased c Fos expression.  Ref: 12208848 Genes Dev, 2002
- Elevated levels of AP 1  [ **JUN** ]  complexes, including significant increases in c  **Jun** , JunB and Fra 1  [ **FOSL1** ] , were found in asbestos exposed RPM cells, but only Fra 1  [ **FOSL1** ]  expression was significantly increased and protracted in both asbestos exposed RPM cells and mesothelioma cell lines.  Ref: 12414630 Cancer Res, 2002
- 1alpha, 25 Dihydroxyvitamin D3 also enhanced the expression of the activator protein 1  [ **JUN** ]  subunits, c Fos, Fra1  [ **FOSL1** ] , and c  **Jun**  as determined by northern and western blotting.  Ref: 12648218 J Invest Dermatol, 2003
- Among several immediate early gene products, only  **Jun**  B and Fra 1  [ **FOSL1** ]  protein levels were increased by LNT, and ginsenoside Rd attenuated  **Jun**  B and Fra 1  [ **FOSL1** ]  protein levels induced by LNT.  Ref: 12785733 Arch Pharm Res, 2003
- ... others, like c  **Jun** , JunB and Fra 1  [ **FOSL1** ]  are essential in embryonic and or postnatal development.  Ref: 12379619 Ann Rheum Dis, 2002
- The formation of the AP 1  [ **JUN** ]  complex is essential, but the rate limiting step is the synthesis of AP 1  [ **JUN** ] , especially Fra  [ **FOSL1** ]  2.  Ref: 12354764 J Biol Chem, 2002
- Immunohistochemical analysis showed that the increase of c Fos and c  **Jun**  proteins by repeated nicotine administrations is mostly medulla specific, while Fra  [ **FOSL1** ]  2 immuno reactivity was shown both in medulla and cortex.  Ref: 12269402 Life Sci, 2002
- Fra 1  [ **FOSL1** ]  and  **Jun**  B immunoreactivities were found in Müller cells.  Ref: 12714663 Invest Ophthalmol Vis Sci, 2003
- Deregulated activation of the IL 6 promoter is for the most part mediated by a combined constitutive activation of the nuclear factor NF kappaB p50 and p65 and the activator protein 1  [ **JUN** ]  AP 1  [ **JUN** ]  JunD and Fra 1  [ **FOSL1** ]  family members as demonstrated by electrophoretic mobility shift assays, site directed mutagenesis, and transfection experiments.  Ref: 12727841 Cancer Res, 2003
- Of the AP 1  [ **JUN** ]  proteins studied, c  **Jun**  was expressed at the highest level, followed by JunD, c Fos, and Fra  [ **FOSL1** ]  2, although different treatments induced slightly different levels of expression.  Ref: 12727447 Mol Cell Neurosci, 2003
- Our results suggest that ginsenoside Rd exerts an inhibitory action against NO production via blocking phosphorylation of ERK, in turn, suppressing immediate early gene products such as  **Jun**  B and Fra 1  [ **FOSL1** ]  in C6 glioma cells.  Ref: 12785733 Arch Pharm Res, 2003
